# Enolase of *Staphylococcus lugdunensis* Is a Surface-Exposed Moonlighting Protein That Binds to Extracellular Matrix and the Plasminogen/Plasmin System

**DOI:** 10.3389/fmicb.2022.837297

**Published:** 2022-03-03

**Authors:** Muzaffar Hussain, Christian Kohler, Karsten Becker

**Affiliations:** ^1^Institute of Medical Microbiology, University Hospital Münster, Münster, Germany; ^2^Friedrich Loeffler Institute of Medical Microbiology, University Medicine Greifswald, Greifswald, Germany; ^3^Interdisciplinary Centre for Clinical Research (IZKF), University Hospital Münster, Münster, Germany

**Keywords:** adhesion, enolase, glycolysis, moonlighting proteins, plasminogen plasmin system, *Staphylococcus lugdunensis*, coagulase-negative staphylococci, extracellular matrix proteins

## Abstract

The coagulase-negative staphylococcal (CoNS) species *Staphylococcus lugdunensis* is unique in causing serious infections in humans that resemble those of *Staphylococcus aureus* rather than those of other CoNS species. The colonization and invasion of host tissue presupposes the presence of adherence factors, but only a few proteins mediating adhesion of *S. lugdunensis* to biotic surfaces are known yet. Here, we report on the functionality of the *S. lugdunensis* enolase (SlEno), which performs two distinct roles, first, as the metabolic enzyme of the glycolysis, and second, as an adherence factor to the extracellular matrix (ECM) of cells. Phylogenetic analyses of the SlEno confirmed their high conservation to enolases of other species and revealed a closer relationship to *Staphylococcus epidermidis* than to *S. aureus*. Using matrix-assisted laser desorption/ionization time of flight mass spectrometry and Western blot experiments, we identified SlEno to be located in the cytoplasm as well as on the cell surface of *S. lugdunensis*. Recombinantly generated and surface-associated SlEno showed the usual enolase activity by catalyzing the conversion of 2-phosphoglycerate to phosphoenolpyruvate but, in addition, also displayed strong binding to immobilized laminin, fibronectin, fibrinogen, and collagen type IV in a dose-dependent manner. We also showed a strong binding of SlEno to plasminogen (Plg) and observed a tissue plasminogen activator (tPA)-dependent conversion of Plg to plasmin (Pln) whereby the Plg activation significantly increased in the presence of SlEno. This interaction might be dependent on lysines of the SlEno protein as binding to Plg was inhibited by ε-aminocaproic acid. Furthermore, the enhanced activation of the Plg/Pln system by SlEno enabled *S. lugdunensis* to migrate through a fibrin matrix. This migration was about 10-fold higher than without exogenously added SlEno. Finally, we observed a significantly higher clearance of *S. lugdunensis* by freshly prepared granulocytes and in the presence of anti-SlEno antibodies. In conclusion, these data demonstrate for the first time a moonlighting function of the *S. lugdunensis* enolase, which is an underrated virulence factor for colonization and invasion of tissues. Hence, SlEno might be a potential vaccine candidate to prevent severe infections caused by this pathogen.

## Introduction

*Staphylococcus lugdunensis* belongs to the group of coagulase-negative staphylococci (CoNS); however, it has a special position among all other CoNS (Frank et al., 2008; Becker et al., 2014; Heilmann et al., 2019). While this opportunistic pathogen is part of the human microbiota colonizing miscellaneous skin surface habitats (van der Mee-Marquet et al., 2003; Kaspar et al., 2016), it is known to cause severe infections, which resemble those caused by *Staphylococcus aureus* rather than “classical” CoNS infections (Etienne et al., 1989; Frank et al., 2008; Böcher et al., 2009; Ravaioli et al., 2012; Seng et al., 2017). In particular, highly aggressive courses of infective endocarditis in native and prosthetic valves with high mortality similar to *S. aureus* have been published (Vandenesch et al., 1993; Patel et al., 2000; Jones et al., 2002; Anguera et al., 2005).

Despite its pathogenic capacity and clinical impact, the mechanisms of *S. lugdunensis’* pathogenicity are still unclear. Adherence to cell or tissue surfaces is the first step to initiate the colonization and invasion of the host tissue. In *S. lugdunensis*, several cell wall-binding proteins were described as important adherence determinants, such as the von Willebrand factor-binding protein (vWbf), the fibrinogen-binding surface protein (Fbl) of *S. lugdunensis*, the *S. lugdunensis* autolysin (AtlL), and the *S. lugdunensis* surface proteins (SlgA/E/G) (Mitchell et al., 2004; Nilsson et al., 2004; Heilbronner et al., 2011; Hussain et al., 2015; Liesenborghs et al., 2016). Other virulence factors involved, e.g., in biofilm formation have also been described (Rajendran et al., 2015; Lebeurre et al., 2019). Recently, the significance of the *S. lugdunensis* housekeeping sortase (SrtA) to anchor cell surface proteins by facilitating the adherence to eukaryotic cell structures has been clarified (Hussain et al., 2020). For many staphylococcal species, the possession of numerous proteins that have multiple functions have been described (Geoghegan and Foster, 2015). However, no data are available on the impact of those proteins, also known as moonlighting proteins (Jeffery, 1999), for *S. lugdunensis* particularly for the attachment to the extracellular matrix (ECM) and plasminogen.

Over the past two decades, it became evident that many proteins of microorganisms are multifunctional whereby a single protein performs multiple independent functions due to the use of different regions of the protein structure (Jeffery, 2020; Hemmadi and Biswas, 2021; Rodriguez-Saavedra et al., 2021). Those moonlighting proteins have very versatile functions. They are often key enzymes of central metabolic pathways and simultaneously are important virulence determinants in many species (Singh and Bhalla, 2020). It was hypothesized that their high expression and high structural conservation toward their host counterparts employs those proteins as virulent factors because the host immune system may elicit an insufficient protective action against these invading bacterial virulence factors (Franco-Serrano et al., 2018; Singh and Bhalla, 2020; Hemmadi and Biswas, 2021; Rodriguez-Saavedra et al., 2021). In addition, several proteins with known moonlighting functions are associated with human diseases like cancer and autoimmune disorders (Zhao, 2019; Singh and Bhalla, 2020; Pirovich et al., 2021).

In general, moonlighting proteins are mainly highly conserved housekeeping proteins involved in chaperone function, stress response, or metabolism. In addition to the enzymes of the tricarboxylic acid cycle (TCA), the glycolytic enzymes are among the most abundant bacterial moonlighting proteins. Presumably up to 7 out of 10 proteins in the glycolytic metabolic pathway have a moonlighting function (Kim and Dang, 2005). Among the glycolytic enzymes, the enolase (phosphopyruvate hydratase E.C. 4.2.1.11) plays a prominent role as a classical moonlighting protein. It belongs to the metalloenzymes and catalyzes the reversible interconversion of 2-phosphoglycerate (2-PG) and phosphoenolpyruvate (PEP). The enolase can act as a heat shock protein, modulates gene transcription and is involved in autoimmune diseases (Pancholi, 2001). Interestingly, it was found as a cell surface adhesin in a variety of microorganisms interacting with the ECM and binding to cytokeratin, salivary mucin, collagen, laminin and/or fibrinogen (Molkanen et al., 2002; Carneiro et al., 2004; Ge et al., 2004; Antikainen et al., 2007; Esgleas et al., 2008; Feng et al., 2009; Kesimer et al., 2009; Boleij et al., 2011; Fulde et al., 2013).

The most common moonlighting function of enolases among pathogenic bacteria appears to be the binding of plasminogen (Plg) (Henderson and Martin, 2011). Generally, the plasminogen/plasmin system displays a striking role in the host defense by fibrinolysis of fibrin clots and represents an essential component to maintain homeostasis and vascular potency (Cesarman-Maus and Hajjar, 2005). However, after hijacking Plg by the enolase on the bacterial cell surface, the Plg is activated by host-derived plasma proteins to plasmin (Pln), whereby enolases further enhance this conversion (Boyle and Lottenberg, 1997; Bergmann et al., 2005; Antikainen et al., 2007; Hemmadi and Biswas, 2021). Thus, the enolase-Pln-surrounded bacteria are able to dissolve fibrin meshwork effectively. Furthermore, Pln degrades the major glycoprotein of basement membranes, i.e., laminin. It activates latent matrix metalloproteinases (MMPs), which, in turn, degrade all main constituents of the ECM, and in addition, Pln modulates inflammation and antibacterial immunity (Parks et al., 2004). In consequence, this Pln-proteolytic activity facilitates pathogen invasion by solving the ECM matrix. Numerous pathogens are known to use the human Plg/Pln system for the migration across host tissue barriers into the host (Boyle and Lottenberg, 1997; Bergmann et al., 2005; Lahteenmaki et al., 2005; Knaust et al., 2007; Hemmadi and Biswas, 2021).

To the best of our knowledge, we describe here for the first time the *S. lugdunensis* enolase (SlEno) as a moonlighting protein that promotes the attachment of this staphylococcal species to eukaryotic cells as a possible virulence factor. We demonstrated the binding of SlEno to five ECM proteins and also showed the activation of Plg by SlEno resulting in enhanced migration through a fibrin matrix. In conclusion, this work provides clear evidence that the enolase of *S. lugdunensis* is a moonlighting protein with multiple functions, one of which is in the central metabolic pathway of glycolysis, while having simultaneously other functions as an adherence and migration factor.

## Materials and Methods

### Bacterial Strains and Growth Conditions

*Staphylococcus aureus* and *Staphylococcus lugdunensis* strains were routinely cultivated aerobically at 37°C in brain heart infusion (BHI) broth or agar (Merck, Darmstadt, Germany), tryptic soy broth (TSB, Difco™, BD Bioscience), or Mueller Hinton broth (Mast, Merseyside, United Kingdom) or agar. *Escherichia coli* cells were grown aerobically at 37°C in lysogeny broth (LB) medium or LB agar. All strains used in this study are shown in Table 1.

**TABLE 1:** Bacterial strains used in this study.

Strains	Relevant genotype or plasmid	Properties	Reference or source
**Staphylococcal strains**			
*S. lugdunensis* Sl20, Sl44, Sl48, Sl105, Sl241, Sl252, and Sl253		Clinical isolates	Sweden^a^
*S. lugdunensis* Sl44		Clinical isolate	Germany^b^
*S. aureus* 6,850	*spa* type t185; sequence type 50 [ST50]	Clinical isolate	GenBank accession number CP006706
***E. coli* strains**			
TG1	*supE hsdΔ5 thiΔ(lac-proAB) F’(traD36 proAB^+^ lacI^q^ lacZΔM15)*	Cloning host	Stratagene
TG1 (pQEno)	pQE30*Eno*	Cloning *Eno*	This study

*^a^Kindly provided by G. Kahlmeter (Växjö, Sweden).*

*^b^Kindly provided by F. Szabados and S. Gatermann (Bochum, Germany).*

### Cell Surface, Cell Wall, and Whole Cell Protein Preparations

*Staphylococcus lugdunensis* strains were grown for 18 h in BHI at 37°C with 160 rpm; after centrifugation (5,000 × *g*, 4°C), the pellet was washed once with PBS and suspended in 1 M LiCl and stirred for 1 h at 37°C and pelleted (6,000 × *g*, 4°C). The supernatant was desalted on Nap-25 column as described before (Hussain et al., 1999). For the SDS extraction method, the pellet was resuspended in the extraction buffer [125 mM Tris–HCl (pH 7.0) plus 2% SDS; Sigma-Aldrich Chemie GmbH, Deisenhofen, Germany], heated at 95°C for 3 min, and then centrifuged at 5,000 × *g* for 3 min (Hussain et al., 2001). For the preparation of whole-cell lysate, bacteria were suspended in Tris–HCl buffer [50 mM Tris, 150 mM NaCl (pH 8.0)] with recombinant lysostaphin (Applied Micro, New York, NY, United States) along with lysozyme (Merck, Darmstadt, Germany) containing a protease inhibitor cocktail [1 mM phenylmethylsulfonyl fluoride (Sigma), 2 mM N-ethylmaleimide (Sigma), and 1 mM EDTA (Sigma), final concentrations] (Hussain et al., 1999). After 30 min of incubation at 37°C, the mixture was centrifuged (5,000 × *g*, 4°C) for 10 min, and the liquid supernatant was used for further experiments. For the isolation of the cell wall fraction, cultures of *S. lugdunensis* from 50 ml of cultures were harvested by centrifugation (4,000 × *g*, 10 min), washed in PBS, and the pellets were resuspended in 3.5 ml of digestion buffer [50 mM Tris–HCl, 20 mM MgCl_2_, 30% (wt/vol) raffinose; pH 7.5] containing complete mini-EDTA-free protease inhibitors (Roche, Germany). Cell wall proteins were solubilized by digestion with lysostaphin 300 μl (500 μg ml^–1^) at 37°C for 30 min in 2-ml tubes. Protoplasts were harvested by centrifugation (5,000 × *g*, 15 min), and the supernatant was retained as the cell wall fraction.

### Mass Spectrometric Peptide Mapping and Sequencing Analysis

A protein band at around 52-kDa molecular mass on SDS-PAGE visualized with Coomassie blue stain was subjected to mass spectrometric peptide mapping and sequencing analysis using a commercial service provided by Alphalyse A/S, DK-5220 Odense, Denmark. Briefly, the protein samples were reduced and alkylated with iodoacetamide, i.e., carbamidomethylated, and subsequently digested with trypsin. The resulting peptides were concentrated on a ZipTip micropurification column and eluted onto an anchorchip target for analysis on a Bruker AutoFlex III MALDI TOF/TOF instrument. The peptide mixture was analyzed in positive reflector mode for accurate peptide mass determination. MALDI MS/MS was performed on 15 peptides for peptide fragmentation analysis, i.e., partial sequencing. The MS and MS/MS spectra were combined and used for database searching using the Mascot software. The protein identification is based on a probability-scoring algorithm^1^, and the significant best matching protein is shown in the result report. Homologous proteins with a lower score were not included.

### Subcellular Localization of Enolase

To determine the distribution of enolase in different bacterial compartments, the LiCl extract containing the cell wall fraction and the whole-cell lysate were separated by SDS page on 10% polyacrylamide gels. The proteins were transferred to nitrocellulose membranes, and the membranes were blocked with 5% skimmed milk for 1 h at room temperature. Afterward, they were washed twice with TBST, the membranes were incubated with anti-*S. lugdunensis* Eno Abs for 1 h at room temperature. The membranes were washed three times with TBST and then incubated with AP-conjugated anti-rabbit Ab at room temperature for 1 h. After washing three times, the membranes were developed with nitroblue tetrazolium/5-bromo-4-chloro-3-indolylphosphate (NBT/BCIP) color reaction.

### Purification of Native Enolase (SlEno)

*Staphylococcus lugdunensis* strains were grown overnight in BHI at 37°C with 160 rpm. After centrifugation (5,000 × *g*, 4°C) of the culture, the pellet was washed once with PBS and suspended in 1 M LiCl and stirred for 1 h at 37°C. After an exchange of LiCl with PBS on a Nap-25 column (GE Healthcare), the extract was loaded on a High-Q resin column (Bio-Rad). After washing with PBS, the column was eluted stepwise by increasing the concentrations of NaCl (0.25 M/0.5 M/0.75 M) in PBS. Enolase was eluted in fractions with 0.75 M NaCl in PBS. The NaCl in SlEno preparation was removed on Nap-25 column, and the native enolase was stored at –20°C in PBS.

### Cloning, Expression, and Purification of *Staphylococcus lugdunensis* and Human Recombinant Enolases

A set of primers consisting of ForEnolBamHI 5′CTC GGA TCC ATG CCA ATT ATT ACA GAT GTT TAT GCT CGC G 3′ and RevEnolKpnI 5′CTC GGT ACC TTA TTT TTT GAA ACG TCT AAA TTG TAG 3′ was used to amplify the enolase gene from the genomic DNA of *S. lugdunensis* 105 by PCR. The gene was ligated into pQE30 vector (Qiagen) and transformed into *E. coli* TG1 cells. Cells were cultivated with 100 μg/ml of ampicillin until an OD_578nm_ of 0.5, and the enolase expression was induced in the presence of 1 mM isopropyl β-D-1-thiogalactopyranoside (IPTG) for 5 h at 37°C. Afterward, the enolase was purified by affinity chromatography on NI NTA resin under native conditions as described elsewhere (The QIAexpressionist, Qiagen). The bacterial recombinant enolases were used immediately or stored at –20°C until further use. Human enolase (HEno) was acquired from Abcam.

### Generation of Polyclonal Antibodies Against Human and Bacterial Enolases

A standard 70-day procedure was used to prepare polyclonal antibodies in rabbits separately against formalin-fixed whole cells of *S. lugdunensis*, recombinant HEno (rHEno), and recombinant enolase of *S. lugdunensis* (rSlEno), respectively. The immunization procedure was carried out by Genosphere Biotechnologies (France), which afterward provided the polyclonal IgG fractions.

### Evaluation of Cross-Reaction Between Human and Bacterial Enolases

A total of 5 μg of enolases or bovine serum albumin was dropped onto a nitrocellulose filter (Schleicher & Schuell, Keene, NH, United States). After air drying, the filters were blocked with 5% skimmed milk in TBST buffer for 1 h at 37°C, followed by incubation with polyclonal IgG antibodies produced against human or bacterial enolase. AP-conjugated anti-rabbit Ab obtained from goats was used as a secondary antibody (Ab) and detected by NBT/BCIP staining. In a further method, the microtiter plate was coated overnight with 100 μl of purified rHEno1 or rSlEno (2 μg/ml) in PBS at 4°C overnight. Plates were washed with PBST, blocked with 5% skimmed milk for 1 h at 37°C. Of bacterial anti-Eno Abs (anti-SlEno) or human anti-Eno Abs (anti-hEno1), 100 μl in different dilutions was added. Wells were washed twice with TBS-T, and AP-conjugated anti-rabbit Ab obtained in goat (1:1,000 dilution) was added as secondary Ab for 1 h at 37°C. The plates were washed and incubated with 100 μl of alkaline phosphatase substrate and monitored at 405 nm.

The binding of the rSlEno with the rHEno1 was detected by incubation of rHEno1 immobilized on microtiter plates with increasing concentrations of rSlEno following incubation for 60 min. After several washing steps with PBST, anti-rSlEno rabbit antibodies were used as primary antibodies and AP-conjugated anti-rabbit goat antibodies as detection antibodies.

### Determination of Enolase Activity

The enolase activity was determined by measuring the transformation of NADH + H^+^ to NAD^+^ applying the enzymatic assay of enolase (EC 4.2.1.11) by Sigma (Saint Louis, MO, United States) according to the recommendation of the manufacturer^2^.

### Binding of Recombinant Enolase of *Staphylococcus lugdunensis* to Immobilized Fibrinogen, Fibronectin, Laminin, Collagen, and Plasminogen

ELISA plate binding assay was used to determine the binding activity of rSlEno to Fg (Calbiochem), Fn (Chemicon, Temecula, CA, United States), Ln (Sigma), Cn IV (Sigma), and Plg (Sigma). Fg, Fn, Ln, Cn, and Plg (2 μg/ml) were dissolved in PBS, and 2 μg was immobilized in wells of microtiter plates overnight at 4°C. After washing with PBST three times, 2.5 μg of diluted rSlEno was added to the wells for 1 h at 37°C. After repeated washing with PBST, the wells were incubated with 100 μl of 1:1,000 diluted anti-SlEno Ab for 1 h at 37°C. After washing with PBST (two times), 10 μl of 1:1,000 diluted AP-conjugated anti-rabbit Ab were added and incubated for 1 h at 37°C. Finally, all wells were washed three times with PBST and incubated with 100 μl of alkaline phosphatase substrate. The OD was recorded at 405 nm.

### Plasminogen Activation by Recombinant Enolase of *Staphylococcus lugdunensis* and *Staphylococcus lugdunensis* Cells

The tissue plasminogen activator (tPA)-mediated activation of Plg in the presence of bacteria or rSlEno was measured as described earlier (43). Briefly, microtiter wells were coated with *S. lugdunensis* cells in PBS overnight at 4°C and fixed with ice-cold methanol for 10 min at –20°C. Afterward wells were blocked with 1% BSA in PBS for 1 h at 37°C. Immobilized rSlEno was incubated with Plg (1 μg) and 0.45 mM chromogenic substrate (D-valyl-L-lysyl-p-nitroaniline hydrochloride) in the presence of 2 ng of tPA in a final volume of 20 μl. Plasmin activity was assessed at intervals of 1.5 min for 15 min by measuring absorbance at 405 nm. Well-bound *S. lugdunensis* were incubated with Plg (1 μg) and 0.45 mM chromogenic substrate (D-valyl-L-lysyl-p-nitroaniline hydrochloride) in the presence of 2 ng of tPA with/without 1 mM of the known Plg inhibitor ε-aminocaproic acid (EACA; Sigma Aldrich) in a final volume of 200 μl.

### Binding of Plasminogen to Recombinant Enolase of *Staphylococcus lugdunensis* and Recombinant Human Eno1 Protein

The experiments were done using two different strategies. First, wells of microtiter plates were coated overnight at 4°C with 1 μg of rSlEno or rHEno1 diluted in PBS. After blocking and washing, as described above, different amounts (1.25, 2.5, 5, 10, and 15 μg) of Plg were added to the wells. Then wells were incubated with anti-Plg monoclonal antibodies obtained from mouse (Abcam, United Kingdom). Wells were washed, and AP-conjugated anti-mouse Ab (1:1,000 dilution) was added as a secondary antibody for 1 h at 37°C. The microtiter plates were washed two times with TBS-T and incubated with 100 μl of alkaline phosphatase substrate and monitored at 405 nm. Second, the microtiter plates were coated with 1 μg of Plg diluted in the PBS and incubated overnight at 4°C. The plates were then blocked with 1% BSA in PBS for 1 h followed by three washes with PBST. Then different amounts (0.5, 1.0, 1.5, 2.0, 2.5, and 3 μg) of recombinant rSlEno or rHEno1 in PBS were added to the wells, and the microtiter plates were incubated at 37°C for 1 h. The binding of rSlEno or rHEno1 to Plg was detected using either anti-SlEno or anti-HEno as described above. All experiments were done in triplicate.

### Plasminogen Binding Inhibition Studies

To determine the competitive inhibition of the rSlEno with *S. lugdunensis* cells to Plg, microtiter plates were coated with 1 μg Plg, and increasing concentrations of rSlEno were added for 60 min at 4°C. After washing, formalin-fixed *S. lugdunensis* cells were added to the wells for 60 min at 25°C. Adherence of the *S. lugdunensis* cells to Plg was determined by anti-*S. lugdunensis* Ab as primary Ab and AP-conjugated anti-rabbit Ab as secondary Ab. In a second experiment, Plg-coated microtiter plates were incubated with formalin-fixed *S. lugdunensis* cells previously incubated with anti-SlEno Abs. Binding was measured by the detection of the bacteria by anti-*S. lugdunensis* Abs. AP-conjugated anti-rabbit Abs served as secondary Abs. Extracellular matrix binding protein (Emp) and anti-Emp IgG were used as a negative control. The data were recorded as the reduction in the turbidity of the clot as a quantitative parameter of fibrinolytic activity. Results are presented as fibrinolysis values relative to tPA only without addition of enolase.

### Determination of Fibrinolytic Activity by Tissue Plasminogen Activator and Recombinant Enolase of *Staphylococcus lugdunensis*

In brief, fibrin gel was prepared in a well of a microtiter plate at 37°C by incubation of a mixture containing 25 μg/ml of human fibrinogen, 1 U/ml of thrombin, and Plg (10 μm) in a buffer containing 10 mM imidazole and 150 mM NaCl at pH 7.4 for 45 min. The dissolution of the clots was monitored by the addition of 100 μl of tPA (8 μM) or tPA mixed with increasing amounts of recombinant SlEno applied on the surface of the clot. tPA alone was added as a standard for the calculating capacity of tPA/rSlEno to induce Plg-dependent fibrinolysis. The microplates were incubated in a humid chamber at 37°C and vigorously shaken. The course of clot formation and dissolution was monitored by measuring the light absorbance at 405 nm (Jin et al., 2008).

### Fibrinolysis by Cell Surface Bound Enolase of *Staphylococcus lugdunensis* Cells

*Staphylococcus lugdunensis* cells (1 × 10^9^ CFU) were resuspended in 100 μl of PBS containing 1% human pooled serum and incubated with 20 μg of human Plg (Sigma-Aldrich) for 30 min at 37°C. The bacterial cells were then washed and suspended in PBS-EDTA, and 1 × 10^8^ CFU of the Plg-pretreated cells were incubated at 37°C with 4 μg of human Fg and 0.06 kIU of tPA. Bacterial cells were then pelleted at different time points, and the reactions were stopped with SDS-containing sample buffer. After boiling for 3 min, supernatants were collected and resolved by SDS-PAGE.

### Transmigration Through a Fibrin Matrix

A fibrin matrix was produced on membranes of Transwell cell culture inserts (polycarbonate membranes with 6.5-mm diameter and 3-μm pore size; Costar) by incubating 200 μl of PBS with 1 mg of Plg-depleted human Fg (Calbiochem) and 25 U of thrombin of human plasma (Sigma) overnight at 4°C. *S. lugdunensis* cells (1 × 10^9^ CFU) were resuspended in 100 μl of PBS containing 1% pooled human serum (Sigma Aldrich) and were incubated with 20 μg of human Plg (Sigma-Aldrich) for 30 min at 37°C. After washing in PBS-EDTA two times, the Plg-pretreated bacteria with/without additional rSlEno were applied to the fibrin matrix at a concentration of 2 × 10^7^ CFU per 100 μl of PBS-EDTA, and simultaneously, Plg was activated by adding 0.06 kIU of tPA. Bacterial transmigration from the upper to the lower chamber was quantified by plating serial dilutions of the lower chamber solution on blood agar. Experiments were carried out for up to 3 h, and samples were plated at timed intervals (15, 75, 120, and 180 min). After each time point, the Transwell inserts were replaced into a new well containing PBS-EDTA buffer.

### Bactericidal Assay

We used the method of Baltimore et al. (1977) with modifications of Fontan et al. (2000) to evaluate the opsonic capacity of anti-SlEno polyclonal antibodies. Briefly, human granulocytes were freshly isolated from Na citrate-treated blood of healthy donors. For isolation, dextran sedimentation and density gradient centrifugation applying Ficoll-Paque Plus (GE Healthcare) was used according to the instruction of the manufacturer. Residual erythrocytes were eliminated by hypotonic lysis. Neutrophils were resuspended at a final density of 1 × 10^6^ cells/0.5 ml in RPMI 1640 supplemented with 10% heat-inactivated FCS and immediately used for the experiments (Loffler et al., 2010). Then a mixture of equal numbers (6 × 10^6^) of freshly prepared granulocytes and bacteria from a culture in exponential growth phase was incubated with/without 10% (vol/vol) pooled human serum (PHS) (Sigma Aldrich) (as a complement source) or polyclonal anti-SlEno antibodies and 10% (vol/vol) PHS in 0.5 ml of Dulbecco’s modified Eagle’s medium for 1 h with shaking. Aliquots of 100 μl were withdrawn from the assay mixture immediately, and after 1 h, appropriate dilutions were plated on blood agar plates to determine the number of colony-forming units per milliliter. The comparison took place between samples incubated with or without PHS and PHS + antibodies.

### Determination of Antibodies Against Enolase in the Human Serum

Anti-enolase was detected in in sera obtained from the staphylococcal carriers, patients, and healthy individuals. The wells of microtiter plates were coated for 18 h at 4°C with rSlEno. After washing with PBS-T, the plates were blocked with BSA. Afterward, diluted human serum was added, and the plates were incubated at 37°C for 1.5 h followed by washing with PBS-T and addition of 100 μl of AP-anti-human IgG. Finally, the wells were incubated with alkaline phosphate substrate for 10 min at room temperature in the darkness and the OD at 405 nm was determined.

### Ethics Statement

Collection of human blood and cell isolation (Niemann et al., 2012) were conducted with approval of the local ethics committees (Ethik-Kommission der Ärztekammer Westfalen-Lippe und der Westfälischen Wilhelms-Universität Münster, Az. 2008-034-f-S).

### Statistical Analysis

Using GraphPad Prism 5.02, the results were statistically analyzed either by a one-way or two-way ANOVA in combination with Bonferroni’s *post hoc* test (compare all pairs of column or compare selected pairs of column). Differences with *p*-values ≤ 0.05 were considered as significant and are indicated with asterisks: *p ≤ 0.05, ^**^p ≤ 0.01, and ^***^p ≤ 0.001.

## Results

### Identification of *Staphylococcus lugdunensis* Enolase in Cell Surface Preparations of *Staphylococcus lugdunensis*

**FIGURE 1:**
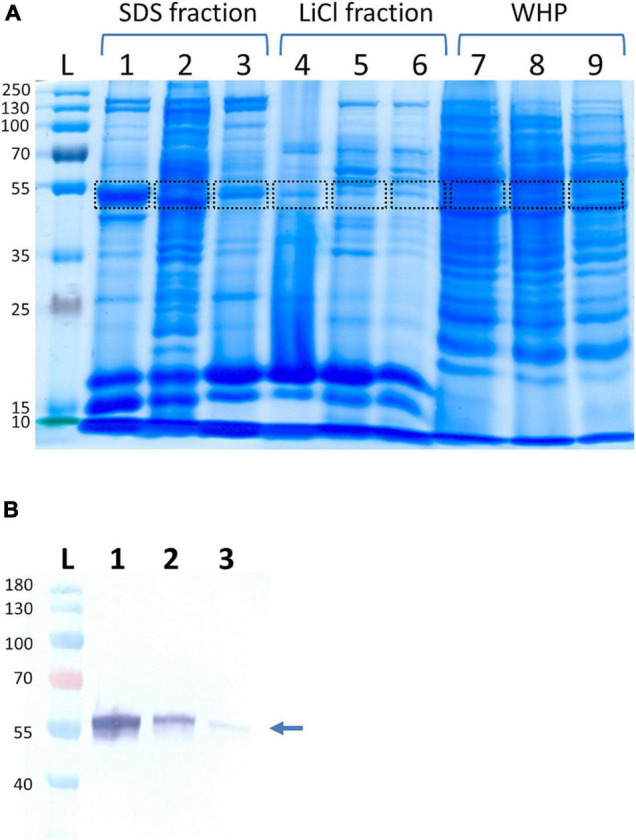
Identification of *Staphylococcus lugdunensis* enolase in different protein extracts. **(A)** *S. lugdunensis* strains Sl20 (lanes 1, 4, 7), Sl44 (lanes 2, 5, 8), and SF48 (lanes 3, 6, 9) were cultivated overnight in brain heart infusion (BHI) medium at 37°C and aerobic condition with a continuously agitation. The bacteria were pelleted by centrifugation, and cell surface-associated proteins were extracted by heating the bacteria in 2% SDS containing sample buffer at 95°C for 3 min (SDS fraction) or stirring in 1 M LiCl (LiCl fraction). Whole-cell proteins (WHP) were generated by lysis of bacterial cells with lysostaphin and lysozyme. Bands were cut and identified by MALDI-TOF-MS/MS. Marked bands correspond to fractions containing the enolase. Lane L, ladder with size information in kDa; lanes 1–3, SDS-extracted cell surface proteins; lanes 4–6, LiCL-extracted cell surface proteins; lanes 7–9, whole-cell proteins. Shown is one out of three biological replicates. **(B)** Western blot analyses of enolase using LiCl generated cell surface protein extracts (1), cell wall extracts (2), and the cytoplasmic protein fraction (3) of *S. lugdunensis*. The respective protein fractions were separated using SDS page, transferred to nitrocellulose membranes and, after blocking the membranes, incubated with anti-SlEno rabbit antibodies. The arrow shows the band corresponding to the enolases.

Since the enolase of *S. lugdunensis* might have a similar function as their counterparts from *S. aureus* and *Streptococcus pneumoniae* (Carneiro et al., 2004; Bergmann et al., 2013), it should also be found on the surface of the bacterial cells. We, therefore, extracted cell surface proteins by two different methods; the LiCl method, an SDS preparation protocol, and finally, we isolated the whole-cell proteins as a control fraction. Using three *S. lugdunensis* strains and applying SDS gel electrophoresis followed by MALDI-TOF MS/MS peptide mass fingerprint and MALDI-TOF/TOF peptide sequencing analysis, a band close to the 55-kDa marker in all three extracted protein fractions was identified as enolase with an MW 47,460 Da and a pI of 4.56 (Mascot score, 1,150 and sequence coverage 61%) (Figure 1A). Additional Western blot analysis of the cytoplasmic, as well as cell surface and cell wall protein fractions, confirmed the high concentration of SlEno on the cell surface of *S. lugdunensis* (Figure 1B).

A protein blast search with the putative protein sequence in the NCBI sequence database^3^ revealed significant homologies to bacterial α-enolases, a family of proteins involved in carbohydrate transport and metabolism. Sequence analysis showed that SlEno contains neither a signal peptide nor an LPXTG motif, which is in concordance to enolases of all other entities. An intra-alignment between two sequenced *S. lugdunensis* strains (N920143 and HKU09-01) showed 100% identity to each other (not shown), and alignments to different staphylococci (*S. epidermidis* RP62A, *S. aureus* 6850, and *S. carnosus*) revealed a very high amino acid consensus of 96.67, 93.08, and 88.72%, respectively. Alignments with other *Firmicutes* members (*Streptococcus pyogenes* MGAS10270391, *Lactobacillus acidophilus* 30SC, and *Bifidobacterium bifidum* PRL2010) revealed lower identities (79.12, 48.02, and 54.81%, respectively) (Supplementary Figure 1).

**FIGURE 2:**
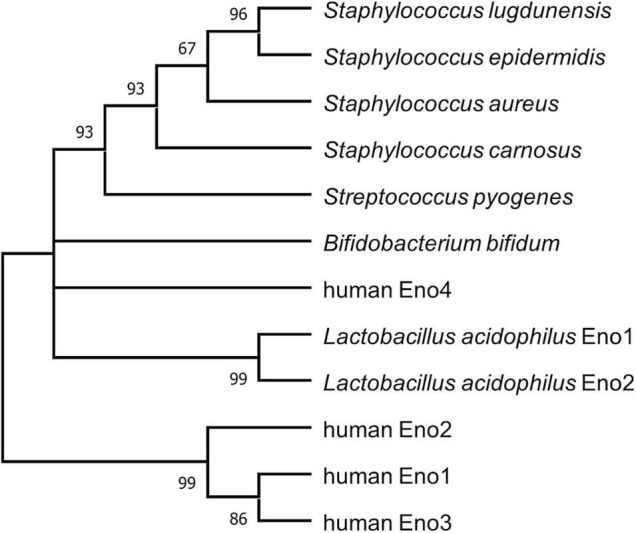
Bootstrap consensus tree inferring molecular phylogenetic of enolase. The maximum likelihood analysis was used to infer the evolutionary relationship for enolases of selected species (Le and Gascuel, 2008). Branches corresponding to partitions reproduced in less than 50% bootstrap replicates are collapsed. The percentage of replicate trees in which the associated taxa clustered together in the bootstrap test (1,000 replicates) are shown next to the branches (Felsenstein, 1985). This analysis involved 12 amino acid sequences. There were a total of 638 positions in the final dataset. Evolutionary analyses were conducted in MEGA X (Kumar et al., 2018).

Four different human enolases (HEno), enolases 1–4 (HEno1-4), are described. HEno1-3 (α-, γ-, and β-enolase) are comprised of 434 amino acids and showed about 50% identities to SlEno (Supplementary Figure 1). HEno4 is much bigger (625 amino acids) than their HEno1-3 paralogs and showed only a low identity of about 26.7% to SlEno (Nakamura et al., 2013). Interestingly, analysis of the amino acid composition especially for lysine, arginine, glutamic acid, and aspartic acid of all used enolase sequences suggest a negative charge for almost all bacterial enolases, but not for the mainly expressed HEno1, which showed no net charge. Only HEno2 and 4 might be negatively charged (Supplementary Table 1). Furthermore, *in silico* sequence analyses revealed a close phylogenetic relationship of SlEno to enolases of other staphylococcal species and *S. pyogenes*, while human enolases and those of *L. acidophilus* and *B. bifidum* were less related (Figure 2 and Supplementary Table 2).

### Evaluation of Cross Reactions Between Anti-*Staphylococcus lugdunensis* Enolase and Anti-human Enolase Antibodies and Determination of Cross Reactions of Sera From Patients With *Staphylococcus aureus* Infections to Recombinant Enolase of *Staphylococcus lugdunensis*

**FIGURE 3:**
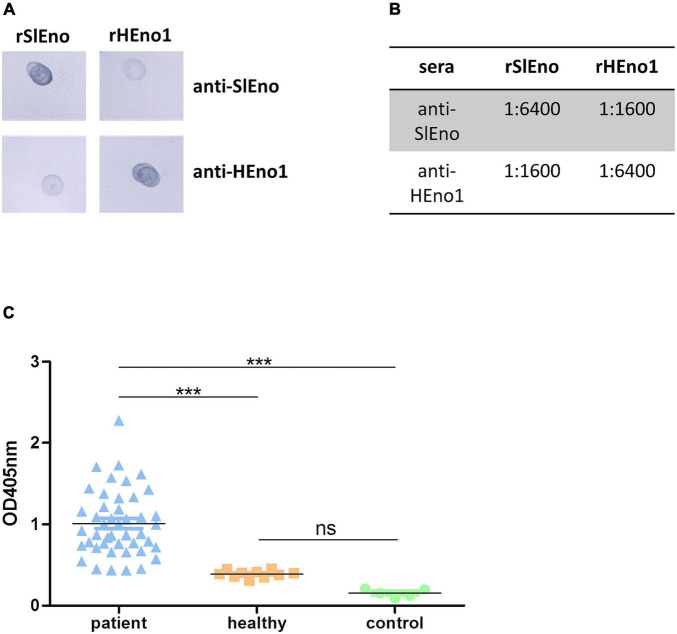
Evaluation of anti-*S. lugdunensis* enolase (SlEno) or anti-human enolase (HEno) antibody cross reactions against recombinant enolase of *S. lugdunensis* (rSlEno), or recombinant HEno (rHEno), and determination of cross reactions to rSlEno by incubation with sera drawn from patients with *Staphylococcus aureus* infections and control sera. **(A)** Of each recombinant enolase [rSlEno–*S. lugdunensis*; recombinant human Eno1 protein (rHENO1)–human enolase 1], 5 μg was spotted on nitrocellulose membrane, blocked, and incubated with 1:10,000 diluted polyclonal anti-SlEno or anti-HEno1 rabbit antibodies. **(B)** Determination of cross reactions between rSlEno and rHEno1 with anti-HEno1 or anti-SlEno rabbit antibodies using an ELISA approach. Of purified rSlEno or rHEno1, 0.2 μg was coated on microtiter plates and incubated with different concentrations of anti-SlEno or anti-hEno1 rabbit antibodies. The dilution factor endpoint was 1:6,400. After incubation with anti-rabbit antibodies, plates were washed, incubated with alkaline phosphatase substrate, and monitored at 405 nm. **(C)** Cross reactions of antibodies against rSlEno were measured using an ELISA approach. To do that, rSlEno was immobilized on microtiter plates. Afterward, the microtiter plates were incubated with sera of patients suffering from infection with *S. aureus* (patient), sera of healthy blood donors (healthy), or PBS as control. Anti-enolase Abs were detected by AP-conjugated anti-human IgG Abs. Statistical analyses were performed using one-way ANOVA with Bonferroni multiple comparisons post-test (***p < 0.001, ns, not significant).

The lower identities to human enolases were examined by cross-reaction experiments using polyclonal antibodies generated with recombinant enolases of *S. lugdunensis* or the human enolase variant. Polyclonal antibodies reacted with purified recombinant HEno1 and rSlEno, suggesting cross-reactions to human and bacterial enolases probably by their structural conservation (Figure 3A). Moreover, antibodies generated with rSlEno showed an about four times higher ELISA titer with the enolase of *S. lugdunensis* then with HEno1 and *vice versa*, which was verified by Western blot experiments (Figure 3B). It is known that a high titer of anti-SaEno antibodies can be found in patients suffering from *S. aureus* infections (Glowalla et al., 2009). Because of the high identity of about 93% of the enolases of *S. aureus* and *S. lugdunensis*, we assume that sera drawn from patients with *S. aureus* infections could also detect the enolase of *S. lugdunensis*. Therefore, we applied rSlEno to microtiter plates and incubated them with sera of *S. aureus* patients or healthy blood donors. As shown in Figure 3C, we detected strong signals in sera from *S. aureus* patients to the enolase of *S. lugdunensis*. In contrast, sera of healthy blood donors or the controls showed significantly lower signals to SlEno.

### Biochemical Characterization of Extracellular *Staphylococcus lugdunensis* Enolase

**FIGURE 4:**
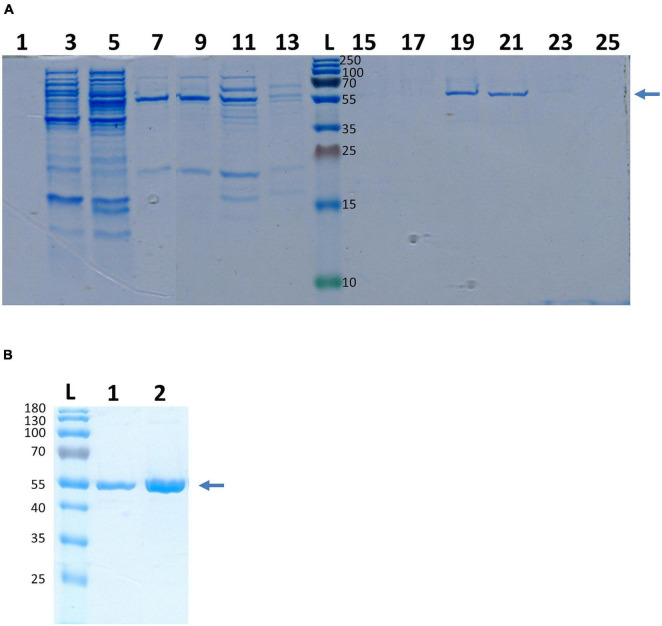
Purification of enolase from *S. lugdunensis* cell surface fraction and recombinant enolase of *S. lugdunensis* and humans. **(A)** Purification of SlEno was realized by ion exchange chromatography. Cell surface fraction was generated by the LiCl treatment and loaded on a high-Q resin column. Increasing concentrations of NaCl (0.25 M/0.5 M/0.75 M) were used for the elution from the column. The fractions (1–25) of the 0.75 M elution step are shown. Gels were stained with Colloidal Coomassie Blue Stain (CCBS). The arrow shows the band corresponding to the enolase. Numbers right of the ladder (L) show kDa of the ladder bands. **(B)** SlEno was cloned with the pQE30 vector and expressed in *Escherichia coli*. Afterward the rSlEno were purified *via* Ni NTA resin, and the elution was separated by SDS page. Recombinant human Eno1 protein (rHEno1) was purchased from a commercial source (Abcam). Gels were stained with CCBS. The arrow shows the band corresponding to the enolases. Numbers on the left show kDa and corresponds to the bands of the ladder (L). 1–rSlEno, 2–rHEno1.

**FIGURE 5:**
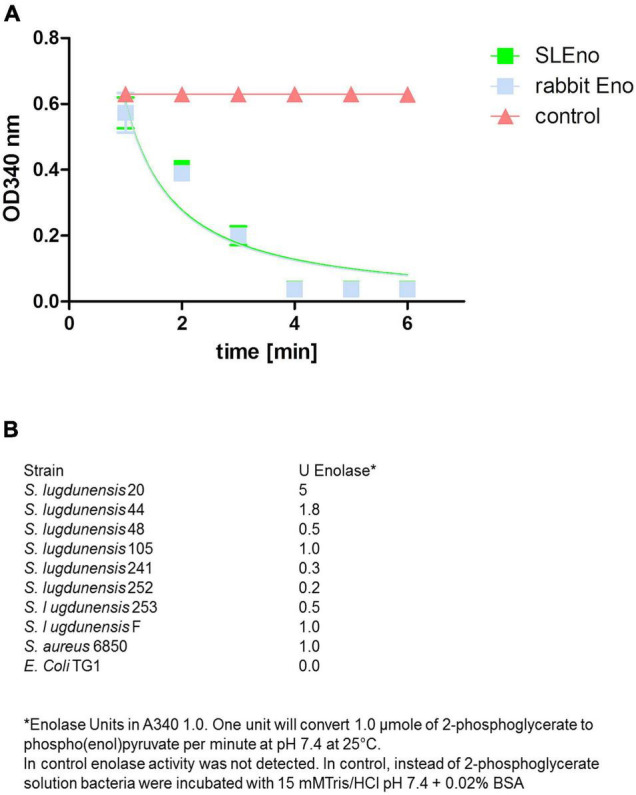
Enzymatic activity of enolases of *S. lugdunensis*, rabbit, and further different species. **(A)** The enzymatic activity of purified SlEno and rabbit enolase was measured over time using a coupled enzyme assay based on the conversion of 2-phosphoglycerate to phosphoenolpyruvate (PEP) followed by the conversion of PEP to pyruvate and last lactate by pyruvate kinase and lactate dehydrogenase. The resulting conversion of NADH + H to NAD + was measured. The amount of NADH oxidized in the above reaction is stoichiometric to the amount of 2-phosphoglycerate. As a control, enolase was omitted. **(B)** Glycolytic activities of enolases were measured in intact, but formaldehyde-fixed cells of different *S. lugdunensis* strains, *S. aureus*, and *E. coli* TG1. Shown are the enolase units.

Actually, enolases are metalloenzymes responsible for the catalysis of the conversion of 2-phosphoglycerate (2-PG) to phosphoenolpyruvate (PEP) during glycolysis. We performed a biochemical characterization to access whether the surface-associated enolase of *S. lugdunensis* still possesses the enzymatic activity of the glycolytic pathway. Therefore, we purified the cell surface-bound enolase by anion-exchange chromatography from LiCl extracts and confirmed the purity by SDS PAGE (Figure 4A). The catalytic activity of SlEno was compared with a commercially available enolase of rabbit muscle as a control. The conversion of 2-phosphoglycerate (2-PG) to phosphoenolpyruvate (PEP) was determined by the combined enzymatic reaction, which last enables the determination of an NAD^+^/NADH + H^+^ ratio by a spectrophotometric assay. The results are summarized in Figure 5A. Both enzymes, the surface originated SlEno and the control rabbit muscle enolase, catalyze the conversion of 2-PGE to PEP quite similar (Figure 5A). The production of PEP increased with time for both enolases and reached maximal concentration at 4 min. In addition, we generated recombinant enolases of *S. lugdunensis* and humans using the pQE30 vector. Both recombinant enolases were expressed in *E. coli* and purified in a single step on Ni-NTA resin as shown in Figure 4B. Quite identical results were obtained when rHEno1 and rSlEno were used in enolase activity enzymatic assays (data not shown). Finally, these data were complemented by results using washed, but intact and formaldehyde fixed, different bacterial species. All used *S. lugdunensis* strains showed enolase activities, although with differences in enzyme kinetics. As expected, we also observed enolase activity on the cell surface of the *S. aureus* 6,850 positive control. No enolase activity was measureable in the negative *E. coli* TG1 control (Figure 5B). Together, these data clearly showed a catalytic activity of SlEno on the cell surface of *S. lugdunensis* cells.

### Enolase of *Staphylococcus lugdunensis* Binds Different Extracellular Matrix Proteins

**FIGURE 6:**
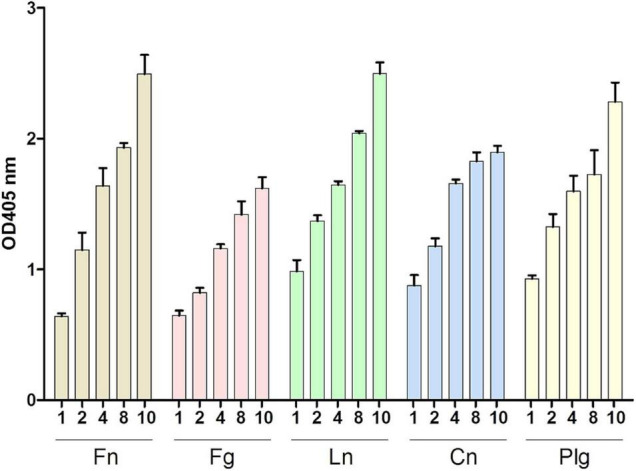
Binding of SlEno to extracellular matrix (ECM) proteins. ECM proteins (fibronectin, Fn; fibrinogen, Fg; laminin, Ln; collagen type IV, Cn; and plasminogen, Plg) were immobilized to microtiter plates and incubated with rSlEno (10 μg/ml) for 2 h. After washing, the rSlEno was detected using anti-SlEno rabbit antibodies as primary and anti-rabbit goat-AP as secondary antibodies. Results are shown as the mean of four independent experiments with the standard deviation (SD).

**FIGURE 7:**
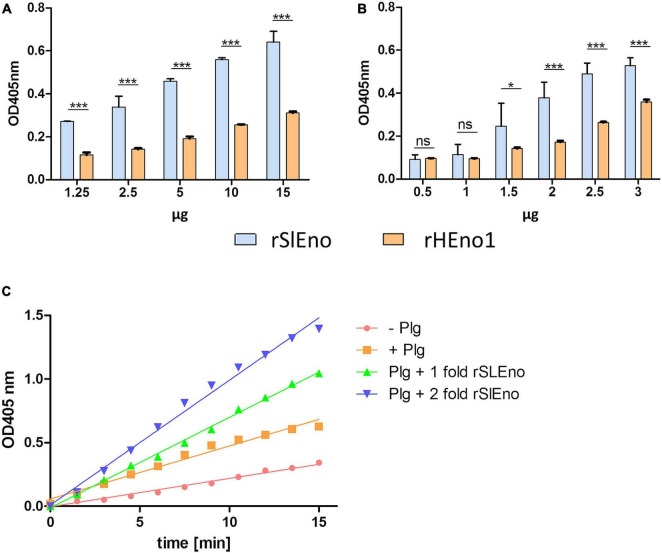
Binding of rSlEno and rHEno1 to plasminogen **(A,B)** and conversion of plasminogen into plasmin **(C)** by rSlEno. **(A,B)** Either 1 μg of rSlEno or rHEno1 **(A)** or Plg **(B)** was immobilized on microtiter plates and incubated with increasing concentrations of Plg **(A)** or rSlEno and rHEno1 **(B)**. Plg was detected by anti-Plg Abs **(A)**, rSlEno and rHEno1 by anti-SlEno or anti-HEno polyclonal Abs **(B)**. Results are shown as the mean of three independent experiments with the standard deviation (SD). Statistical analyses were performed using two-way ANOVA with Bonferroni multiple comparisons post-test (*p < 0.05, ***p < 0.001, ns, not significant). **(C)** rSlEno enhanced the conversion of plasminogen (Plg) into plasmin (Pln) in the presence of tissue tPA. The reaction progress is measurable by the presence of the chromogenic substrate D-valyl-L-lysyl-p-nitroaniline hydrochloride.

**FIGURE 8:**
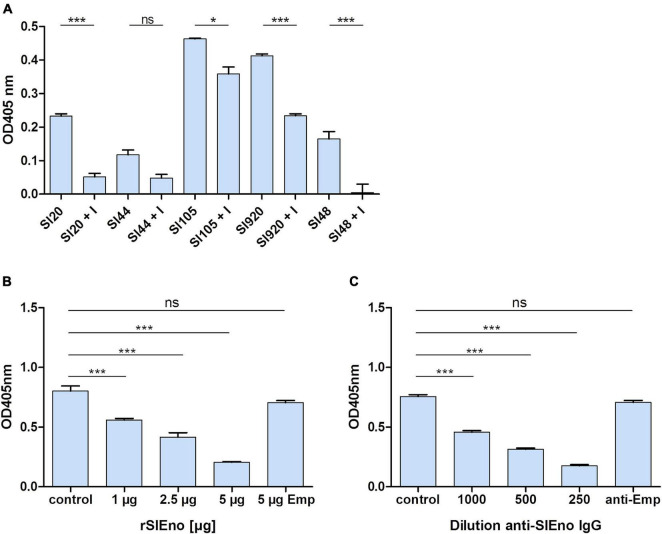
Binding of surface-bound SlEno to plasminogen is inhibitable by ε-aminocaproic acid and inhibition of *S. lugdunensis* binding to plasminogen by rSlEno or anti-rSlEno antibodies. **(A)** The inhibition of enolases by ε-aminocaproic acid (EACA = I) of different *S. lugdunensis* strains coated in microtiter plates was performed in the presence of tPA, D-valyl-L-lysyl-p-nitroaniline hydrochloride, EACA, and Plg. Results are shown as the mean of two independent experiments with the standard deviation (SD). Statistical analyses were performed using one-way ANOVA with Bonferroni multiple comparisons post-test (*p < 0.05, ***p < 0.001, ns, not significant). **(B)** To determine the competitive inhibition of the rSlEno with *S. lugdunensis* cells to Plg, microtiter plates were coated with 1 μg of Plg, and increasing concentrations of rSlEno were added. After washing, formalin-fixed *S. lugdunensis* cells were added to the wells. Adherence of the *S. lugdunensis* cells to Plg was determined by anti-*S. lugdunensis* Abs. Control–no addition of rSlEno, 5 μg Emp–5 μg of the ECM-binding protein extracellular matrix binding protein (Emp) was used as negative control. **(C)** Plg-coated microtiter plates were incubated with formalin-fixed *S. lugdunensis* cells, which were prior incubated with anti-SlEno IgG. Binding was measured by the detection of the bacteria by anti-*S. lugdunensis* IgG. Control–no anti-SlEno IgG was added to the reaction, anti-Emp–negative control using anti-Emp IgG. Results of **(B,C)** are shown as the mean of three independent experiments with the standard deviation (SD). Statistical analyses were performed using one-way ANOVA with Bonferroni multiple comparisons post-test (***p < 0.001, ns, not significant).

As mentioned above, enolases are enzymes important for the glycolytic metabolism of, but play also an important role as, adhesins to the ECM in various pathogens. Here, we tested the ability of SlEno to bind several known bacterial adhesin targets like Fn, Fg, Ln, Cn, and Plg. As shown in Figure 6, the rSlEno binds specifically to immobilized ECM proteins in a dose-dependent manner but quantitatively differently. We found slightly higher binding capacities to Fn, Ln, and Plg than to Cn and Fg. As expected, SlEno failed to interact with immobilized casein used as a negative control (data not shown). The key target for enolases of many pathogens is Plg, which leads to an immobilization of Plg on their cell surfaces and, thus, enhances the activation of Plg to the active serine protease plasmin (Bhattacharya et al., 2012). We determined the capacity of SlEno to bind Plg and to accelerate the conversion of plasminogen to plasmin (Pln) (Figures 7A–C). Both rSlEno and HEno bound Plg and *vice versa*, but the rSlEno revealed a higher binding capacity (Figures 7A,B). To control if the rSlEno leads to a conversion of Plg to Pln, plasminogen was mixed with rSlEno and activation was initiated with the addition of tPA. In the presence of rSlEno, the rate of tPA-dependent plasminogen activation significantly increased compared with incubations without Eno (Figure 7C). We further investigated the interaction between intact *S. lugdunensis* cells and Plg to verify these results. Since conventional genetic manipulation leading to *eno* null mutants is impossible because most moonlighting enzymes from central metabolism are essential enzymes, we proved indirectly that the enolase does indeed promote bacterial adhesion to the ECM or capture plasminogen. Therefore, we performed control experiments with the known Plg inhibitor ε-aminocaproic acid (EACA), a lysine analog that inhibits the capacity of plasminogen to bind to the cell surface through blocking lysine binding sites of Plg. We tested an interaction of the Plg with SlEno as bacterial Plg receptors (Figure 8A). Our data clearly showed that formalin-fixed *S. lugdunensis* cells bind Plg and tPA and that EACA leads to a reduction in the binding capacity of the SlEno to Plg (Figure 8A). The interactions between Plg and *S. lugdunensis* cells were also competitively reduced by the addition of soluble rSlEno (Figure 8B) or polyclonal Abs against the enolase (Figure 8C). Compared with non-enolase binding control Abs anti-Emp IgG, the magnitude of inhibition of Plg interaction was greater in the case of anti-enolase Abs suggesting that the enolase might interact and activate directly Plg on the surface of eukaryotic cells (Figure 8C).

### Enolase Enhances the Fibrinolytic Activity and Transmigration of *Staphylococcus lugdunensis* Through a Fibrin Matrix

**FIGURE 9:**
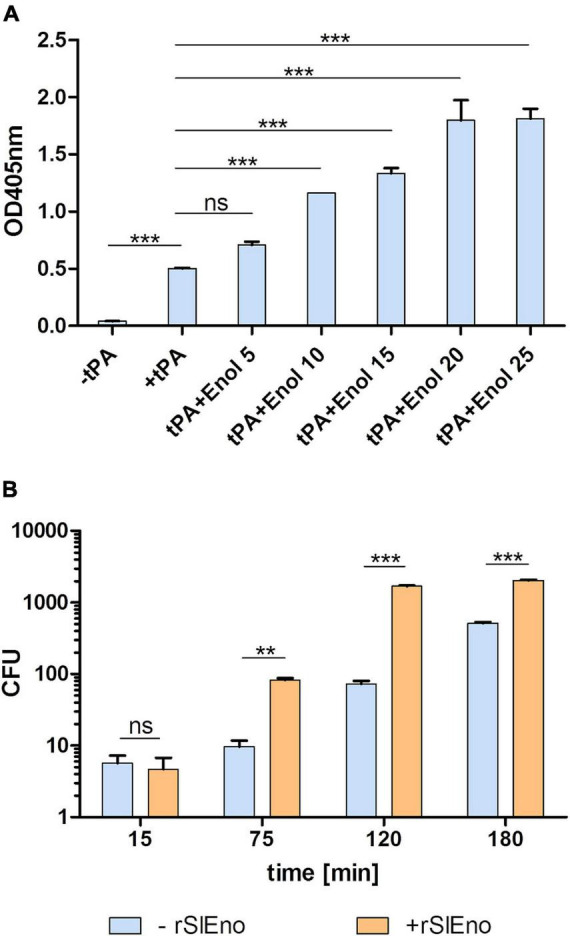
rSlEno triggered the fibrinolytic activity of tissue plasminogen activator (tPA) and enhanced transmigration of *S. lugdunensis* through a fibrin matrix membrane. **(A)** The fibrinolytic activity of rSlEno was determined by an addition of 8 μM tPA and increasing concentrations of rSlEno to microtiter plates containing a fibrin gel. The data were recorded as the reduction in the turbidity of the clot as a quantitative parameter of fibrinolytic activity. Results are presented as fibrinolysis values relative to tPA only without addition of enolase. **(B)** Transmigration of *S. lugdunensis* was determined in Transwell cell culture inserts containing a fibrin matrix. Plg-pretreated bacteria with and without additional rSlEno were applied to the fibrin matrix. After 15, 75, 120, and 180 min, the bacteria that migrated from the upper to the lower chamber were plated on blood agar, and after 24 h of growth at 37°C, the colony-forming units (CFU) were calculated. Results are shown as the mean of three independent experiments with the standard deviation (SD). Statistical analyses were performed using one-way ANOVA **(A)** or two-way ANOVA **(B)** with Bonferroni multiple comparisons post-test (**p < 0.01, ***p < 0.001, ns, not significant).

As shown above, SlEno is able to bind and convert Plg to Pln, which can even be enhanced by the addition of tPA. We therefore assume that Pln degrades fibrin clots (fibrinolysis) and various ECM components, which enable the bacterial migration through tissue barriers. To test this hypothesis, the fibrinolytic capacities of SlEno as well as the plasmin activities were evaluated using fibrinogen-containing jellified matrices. The results suggest that the lysis of fibrinogen was readily promoted upon addition of SlEno. The fibrinolytic activity is enhanced in a dose-dependent manner in the presence of enolase and reaches saturation (Figure 9A). In addition, the incubation of whole human serum, SlEno, and tPA caused a visible degradation of large protein bands to smaller products (Supplementary Figure 2). To make the proof of principle, we tested if an addition of rSlEno leads to an accelerated putative transmigration of *S. lugdunensis* through a fibrin matrix (Figure 9B). The fibrin matrix was generated on membranes of Transwell cell culture inserts. In the presence of Plg and tPA, the number of transmigrated bacteria increased up to 10 CFU per ml in 75 min, but the transmigration significantly increased in the presence of rSlEno. Within the same period of time, the number of transmigrated bacteria grew 10-fold (Figure 9B). However, the partial dissolution of the turbid fibrin matrix in the absence of rSlEno was visible, but the gel matrix completely dissolved after 3 h in the presence of the enolase (data not shown). In sum, these results clearly showed the capacity of rSlEno and cell surface-bound SlEno to enhance the proteolytic degradation of fibrin by activation of Plg to Pln.

### Killing of *Staphylococcus lugdunensis* by Granulocytes in the Presence of Human Sera and Anti-*Staphylococcus lugdunensis* Enolase Antibodies

**FIGURE 10:**
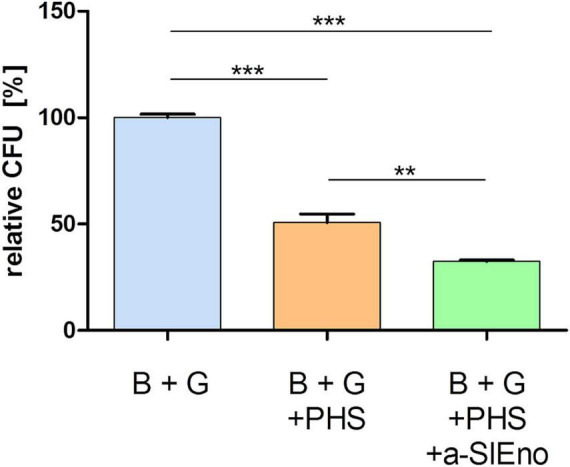
Killing of *S. lugdunensis* by granulocytes in the absence and presence of pooled human sera and anti-SlEno Abs. Exponentially grown *S. lugdunensis* cells (B) and freshly prepared granulocytes (G) were mixed in 0.5 ml of Dulbecco’s modified Eagle’s medium and incubated solely, with pooled human sera (PHS) or PHS plus anti-SlEno antibodies (a-SlEno) for 60 min. Afterward aliquots of bacteria were platted on blood agar plates, and the number of CFUs were determined. Shown are the relative CFUs of B + G + PHS and B + G + PHS + a-SlEno compared with the CFUs of B + G. Results are shown as the mean of three independent experiments with the standard deviation (SD). Statistical analyses were performed using one-way ANOVA with Bonferroni multiple comparisons post-test (**p < 0.01, ***p < 0.001).

Specific antibodies against surface structures of the pathogens are important for the classical activation of the complement system (Merle et al., 2015a,b). We assumed that specific anti-SlEno antibodies could increase the clearance of *S. lugdunensis* by the complement system and freshly prepared granulocytes. To test this hypothesis, we incubated *S. lugdunensis* cells with either granulocytes and pooled human sera (PHS) or granulocytes with PHS and specific polyclonal anti-SlEno IgGs. *S. lugdunensis* cells incubated only with granulocytes served as the control. The addition of PHS causes a significant reduction of around 49% of colony-forming units (CFU) of *S. lugdunensis* 1 h after incubation (Figure 10), but after addition of specific polyclonal anti-SLEno IgGs to the mixtures, we observed a significant reduction of at least 68% of *S. lugdunensis* CFUs (Figure 10), which confirmed our hypothesis.

## Discussion

Enolases belong to the big group of moonlighting proteins, which originally possessed a single function but, through evolution, acquired additional functions (Jeffery, 1999; Antikainen et al., 2007; Hemmadi and Biswas, 2021). In general, most of the known moonlighting proteins are highly conserved enzymes, and enzymes involved in sugar metabolism, in particular, appear to moonlight (Wistow et al., 1988; Yuan et al., 1997; Decker and Wickner, 2006; Huberts and van der Klei, 2010). This phenomenon is well described for bacteria, but is also widespread across all *Eukarya* kingdoms (Decker and Wickner, 2006; Antikainen et al., 2007; Rodriguez-Saavedra et al., 2021). In this study, we describe, for the first time, that the enolase of *S. lugdunensis* functions as a moonlighting protein that may contribute to the pathogenesis of this pathogen.

Enolases are one of the most abundantly expressed cytoplasmic proteins in many organisms. They play a key role in the second half of the Embden–Meyerhof–Parnas glycolytic pathway (Spring and Wold, 1971; Pancholi, 2001). Here, we show that the enolase of *S. lugdunensis* is located in the cytoplasm as well as on the surface (Figures 1, 4). In addition, the SlEno purified from cell surface has still catalytic enolase activity (Figure 5). Hence, we assume similar moonlight functions of the *S. lugdunensis* enolase as already described for other species (Lottenberg et al., 1994; Pancholi, 2001; Bergmann et al., 2005; Antikainen et al., 2007; Itzek et al., 2010; Hemmadi and Biswas, 2021). The location of the enolase on the cell surface is well described, but the secretion principle is not clear because enolases do not have canonical signal peptides or secretion motifs (Ebner et al., 2015). Interestingly, the so-called excretion of cytoplasmic proteins is a common physiological feature in bacteria and eukaryotes (Ebner et al., 2015). In *S. aureus*, the enolase is actively excreted to the cell surface during the exponential growth phase and is not acquired from cells undergoing cell leakage or cell death as proposed by some former theories (Ebner et al., 2016; Hemmadi and Biswas, 2021). A similar excretion process for *S. lugdunensis* is, therefore, very likely. Furthermore, the amino acid identity of the enolases is very high between most species with at least 40–97%, which is in accordance with our results (Supplementary Figure 1; Pancholi, 2001). SlEno is highly identical to the enolases of other staphylococcal species and members of the *Streptococcus* genus. It showed moderate amino acid identities of about 50% to the enolases of the lactate-producing Gram-positive species *L. acidophilus* and *B. bifidum* or even to humans (Supplementary Figure 1). The antibody experiments of our study endorsed this high degree of amino acid identities (Figure 3).

Interestingly, our phylogenetic analysis showed a closer relationship of SlEno to the enolase of *S. epidermidis* than to *S. aureus* or *S. carnosus* (Figure 2 and Supplementary Tables 1, 2). From a phylogenetic viewpoint, *S. lugdunensis* is like *S. aureus* and *S. epidermidis*, part of the *epidermidis–aureus* species group, while *S. carnosus* is part of the more distant *simulans* species group (Lamers et al., 2012). *S. lugdunensis*, *S. epidermidis*, and, in part, also *S. aureus* share, as commensals of the human skin, overlapping environments with fairly similar growth conditions. In contrast, *S. carnosus* has been associated with fermented food and cattle (Becker et al., 2020). Therefore, one could speculate that the core set of enzymes for the basic metabolism including the enolase should be highly adapted and probably greatly identical between species of a genus of the same environments.

Enolases are also found on the cell surface in many eukaryotic and prokaryotic organisms (Antikainen et al., 2007; Hemmadi and Biswas, 2021). Especially, pathogens use enolases to enhance adhesion to different ECM components, thereby increasing the bacterial invasiveness in the host (Miles et al., 1991; Lottenberg et al., 1994; Pancholi and Fischetti, 1998; Pancholi, 2001; Bergmann et al., 2005; Antikainen et al., 2007; Itzek et al., 2010). Here, the SlEno showed increased binding to the ECM proteins fibrinogen, fibronectin, collagen IV, and laminin (Figure 6). This findings are in concordance with earlier studies of enolases of several pathogens like *S. aureus* (laminin and collagen), streptococci (laminin and fibronectin) and also of non-pathogenic lactic acid bacteria (fibronectin, laminin, and collagen) (Carneiro et al., 2004; Antikainen et al., 2007; Castaldo et al., 2009; Li et al., 2015). Interestingly, SlEno binds to all tested ECM proteins, whereas enolases of other species showed variations in their binding capacities (Pancholi, 2001; Antikainen et al., 2007; Hemmadi and Biswas, 2021). This may or may not be a consequence of different techniques, protocols, and/or different ECM protein suppliers used.

Besides adhesion to these classical ECM proteins, SlEno showed also a strong binding of plasminogen (Figures 6, 7). Plasminogen is a liver-derived zymogen circulating in the blood and can be activated to the serine protease plasmin. The activation of Plg to the proteolytic active Pln is driven by different enzymes like the tissue plasminogen activator tPA or the urokinase plasminogen activator (uPA). Many bacteria interact with the Plg system, whereby enolases enhance an activation of Plg by tPA as we also observed for the SlEno (Figures 7, 8; Pancholi, 2001; Lahteenmaki et al., 2005; Hemmadi and Biswas, 2021). It is well known that the binding of Plg to enolases depends on an internal Plg-binding motif like 248-FYDKERKVY-256 as described for pneumococci, but it seems that the basic amino acid lysine elsewhere in enolases is critical for Plg binding in many species (Derbise et al., 2004; Bergmann et al., 2005; Antikainen et al., 2007; Candela et al., 2009; Sha et al., 2009; Serek et al., 2021). The sequence of SlEno contains lysines on the C-terminal as a potential Plg binding site, and the fact that the binding of Plg to the SlEno was hindered by the lysine analog EACA indicates a binding inhibition to the lysines of the SlEno to Plg (Miles et al., 1991; Derbise et al., 2004; Bergmann et al., 2005; Antikainen et al., 2007; Candela et al., 2009; Sha et al., 2009). Further experiments are necessary to specify the crucial lysines in SlEno. However, plasmin dissolves fibrin blood clots, but apart from fibrinolysis, it has a broad spectrum of extravascular functions like the degradation of the ECM (Chapman et al., 1982; Bergmann et al., 2005). Our results suggest that the capture of Plg by SlEno as a cell surface receptor and the tPA-dependent conversion of Plg to plasmin provides the *S. lugdunensis* with proteolytic activity and thereby drives the degradation of fibrin in jellified matrices (Supplementary Figure 2 and Figure 9). This leads to the observed increase in transmigration of *S. lugdunensis* through the jellified matrices and probably to the host ECM. The ECM degradation definitely increases the bacterial invasion into tissue and facilitates invasion and dissemination within the infected host as already has been shown for other pathogens (Pancholi and Fischetti, 1998; Bergmann et al., 2005; Lahteenmaki et al., 2005; Hemmadi and Biswas, 2021). Whereas we proved the binding of SlEno to different ECM proteins using rather indirect methods, Gani et al. (2021) applied two different genetic manipulation strategies (overexpression and a siRNA strategy) to show a binding of the glyceraldehyde 3-phosphate dehydrogenase (GAPDH) of *Mycobacterium tuberculosis* with ECM matrix and the ability to recruit plasmin(ogen) (Gani et al., 2021). Thus, new molecular methods should be evolved to analyze further moonlighting candidates in *S. lugdunensis* and are currently under intensive investigation.

Once inside the host, the bacteria are attacked by the innate and/or adaptive immune systems. A key role in the defense against extracellular pathogens is played by the complement system as part of the innate immunity. It mediates the recognition of pathogens and leads to their clearance by lysis and/or opsonization of phagocytic cells (Merle et al., 2015a,b). A part of the classical activation of the complement system is the recognition of pathogen-specific antibodies of the IgM and/or IgG class (Merle et al., 2015a,b). We showed that specific anti-SlEno IgG antibodies increased the clearance of *S. lugdunensis* by granulocytes in the presence of pooled human serum (Figure 10). These results indicate that anti-rSlEno antibodies recruited by bacteria promote their association with professional phagocytes and their opsonization. Moreover, it implies that the SlEno might be a suitable vaccine candidate against *S. lugdunensis* infections. Several studies with enolases from pro- and eukaryotic pathogens administered as vaccines demonstrated promising results (Arce-Fonseca et al., 2018; Li et al., 2020; Thu Nguyen et al., 2020; Yang and Kim, 2021). However, other groups found only weak potential for the enolase as a potent vaccine (Glowalla et al., 2009; Dumesnil et al., 2019). Therefore, further studies are warranted to clarify this aspect for *S. lugdunensis*.

## Conclusion

Our data indicate that the enolase from *S. lugdunensis* has a very similar moonlight character to enolases described for other pathogens. It combines enzymatic and virulence-associated functions, namely, the conversion of 2-phosphoglycerate to phosphoenolpyruvate and, extracellularly, the interaction with ECM and the plasminogen/plasmin system, which results in an increased adhesiveness. We further conclude that the *S. lugdunensis* Plg/plasmin interaction may represent an important proteolytic system for the spread of this pathogen inside the host and to cause skin and soft-tissue lesions, bacteremia, and infective endocarditis. Therefore, further studies are needed to understand the exact role of this moonlight system in *S. lugdunensis* infections.

## Data Availability Statement

The original contributions presented in the study are included in the article/Supplementary Material, further inquiries can be directed to the corresponding author.

## Ethics Statement

The studies involving human participants were reviewed and approved by the Ethik-Kommission der Ärztekammer Westfalen-Lippe und der Westfälischen Wilhelms-Universität Münster. Written informed consent for participation was not required for this study in accordance with the national legislation and the institutional requirements.

## Author Contributions

MH and KB: conceptualization. MH and CK: data analysis, validation, and visualization. MH, CK, and KB: writing – original draft preparation, writing, review, and editing. KB: supervision and funding acquisition. All authors have read and agreed to the published version of the manuscript.

## Conflict of Interest

The authors declare that the research was conducted in the absence of any commercial or financial relationships that could be construed as a potential conflict of interest.

## Publisher’s Note

All claims expressed in this article are solely those of the authors and do not necessarily represent those of their affiliated organizations, or those of the publisher, the editors and the reviewers. Any product that may be evaluated in this article, or claim that may be made by its manufacturer, is not guaranteed or endorsed by the publisher.

## Footnotes

1
www.matrixscience.com

2
https://www.sigmaaldrich.com/deepweb/assets/sigmaaldrich/product/documents/346/393/e6126enz.pdf

3
http://www.ncbi.nlm.nih.gov/BLAST/
