# Supplementary material for: Enolase of Staphylococcus lugdunensis Is a Surface-Exposed Moonlighting Protein That Binds to Extracellular Matrix and the Plasminogen/Plasmin System
Source: Front Microbiol. 2022 Mar 3;13:837297. doi: 10.3389/fmicb.2022.837297 (PMC8928124; doi:10.3389/fmicb.2022.837297)
Supplement: Supplementary file 1 [file Data_Sheet_1.PDF]

Table S1. Amino acid composition analysis for lysine, arginine, glutamic acid and aspartic acid of enolases of different species.

	D[a]	E[a]	K[a]	R[a]	DE-[a]	KR+[a]	DEKR[a]	KR-DE[a]	KR-DE rel [%]
Staphylococcus lugdunensis	29	44	27	15	73	42	115	-31	-7,1
Lactobacillus acidophilus Eno1	29	39	34	17	68	51	119	-17	-4,0
Lactobacillus acidophilus Eno2	33	39	30	14	72	44	116	-28	-6,5
Bifidobacterium bifidum	29	38	24	20	67	44	111	-23	-5,3
Streptococcus pyogenes	27	40	26	19	67	45	112	-22	-5,1
Staphylococcus carnosus	32	39	30	14	71	44	115	-27	-6,2
Staphylococcus epidermidis	28	44	26	15	72	41	113	-31	-7,1
Staphylococcus aureus	29	41	25	15	70	40	110	-30	-6,9
human Eno 1	26	29	38	17	55	55	110	0	0,0
human Eno 2	30	34	26	20	64	46	110	-18	-4,1
human Eno 3	26	27	37	17	53	54	107	1	0,2
human Eno 4	22	57	47	17	79	64	143	-15	-2,4

[a] – Amino acid composition analysis conducted by MEGA X. Number of the respective amino acid in the protein sequence is shown. K, lysine; R, arginine, E, Glutamic acid, D, aspartic acid , DE- negative charged D and E together , KR+ positive charged K and R together, KR-DE – difference of the number of KR to DE , rel – difference of the number of KR to DE relative to the total number of amino acids of the enolase per species

Table S2. Estimates of evolutionary divergence between sequences of enolases.

	SLG	LAC Eno1	LAC Eno2	BBI	SPY	SAC	SEP	SAU	hEno1	hEno2	hEno3	hEno4
SLG												
LAC Eno1	0.71											
LAC Eno2	0.67	0.29										
BBI	0.55	0.64	0.61									
SPY	0.22	0.73	0.70	0.54								
SAC	0.10	0.73	0.72	0.57	0.23							
SEP	0.03	0.71	0.68	0.57	0.22	0.10						
SAU	0.06	0.71	0.68	0.54	0.20	0.11	0.06					
hEno1	0.65	0.68	0.72	0.62	0.71	0.67	0.65	0.67				
hEno2	0.66	0.66	0.70	0.60	0.68	0.67	0.68	0.69	0.18			
hEno3	0.66	0.72	0.76	0.64	0.70	0.65	0.66	0.66	0.17	0.18		
hEno4	1.30	1.33	1.34	1.32	1.30	1.25	1.30	1.29	1.33	1.30	1.31	

The number of amino acid substitutions per site from between sequences are shown. Analyses were conducted using the Poisson correction model [1]. This analysis involved 12 amino acid sequences. All ambiguous positions were removed for each sequence pair (pairwise deletion option). There were a total of 638 positions in the final dataset. The analyses were conducted in MEGA X [2]

SLG - *Staphylococcus lugdunensis* , LAC Eno1 - *Lactobacillus acidophilus* Eno1 , LAC Eno2 - *Lactobacillus acidophilus* Eno2 , BBI - *Bifidobacterium bifidum* , SPY - *Streptococcus pyogenes* , SCA - *Staphylococcus carnosus* , SEP - *Staphylococcus epidermidis* , SAU - *Staphylococcus aureus* , hEno1-4 - *human* enolases 1 to 4

1. Zuckerkandl E. and Pauling L. (1965). Evolutionary divergence and convergence in proteins. Edited in Evolving Genes and Proteins by V. Bryson and H.J. Vogel, pp. 97-166. Academic Press, New York.
2. Kumar S., Stecher G., Li M., Knyaz C., and Tamura K. (2018). MEGA X: Molecular Evolutionary Genetics Analysis across computing platforms. Molecular Biology and Evolution 35:1547-1549.

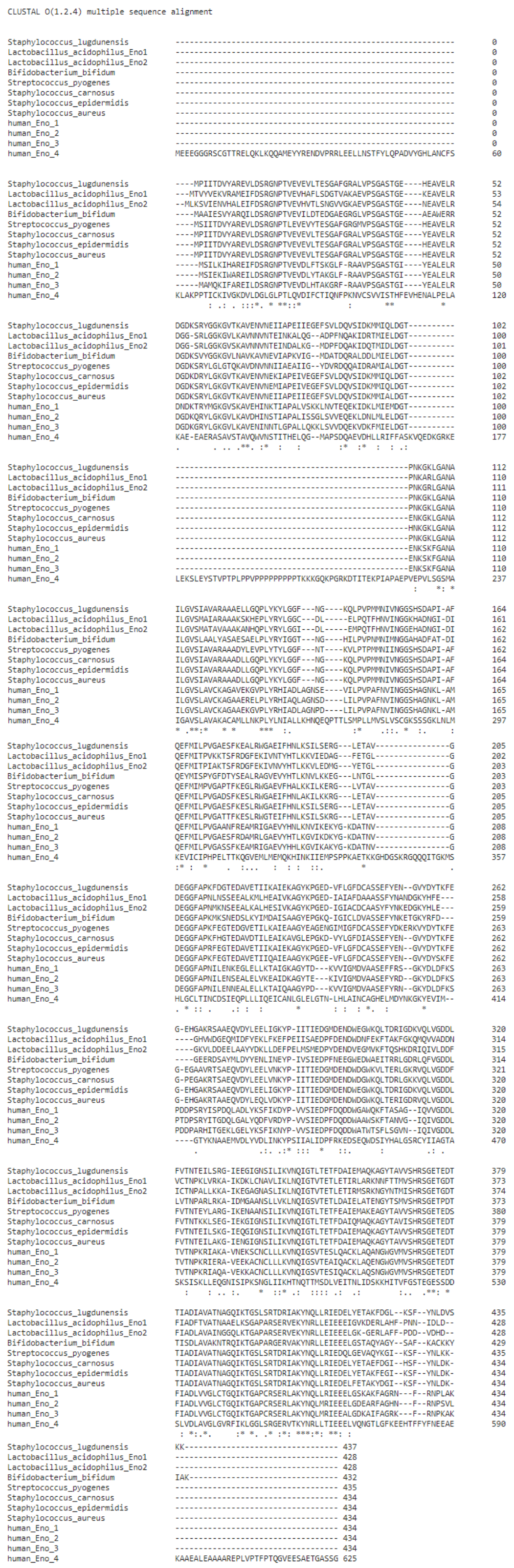

Figure S1. Alignment of SlEno with different bacterial species and human enolases. A "*" indicates perfect alignment, ":" indicates a site belonging to group exhibiting strong similarity and "." indicates a site belonging to a group exhibiting weak similarity.

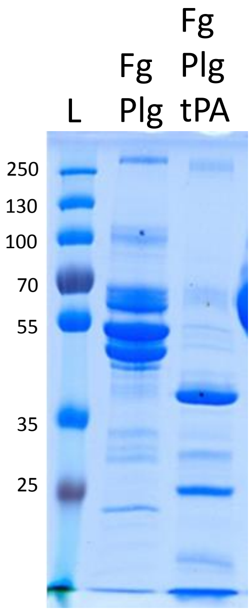

Figure S2. Fibrinolysis is activated by tPA and *S. lugdunensis* cells. Degradation of large protein bands to smaller products are visible in line 3. Cells of *S. lugdunensis* were preincubated with pooled human sera and human plasminogen (Plg) for 30 min at 37 °C. Afterwards the bacteria were washed and incubated with human fibrinogen and tPA. Reactions were stopped by centrifugations and afterwards addition of SDS sample buffer. Then the supernatants were applied to SDS-page and stained with Colloidal Coomassie blue stain. Shown is one experiment out of three. L – ladder, Fg – fibrinogen, Plg – plasminogen, tPA – tissue plasminogen activator
